# Metal in Mandible

**DOI:** 10.11604/pamj.2016.23.192.9425

**Published:** 2016-04-15

**Authors:** Prashanth Panta, Uday Shankar Yaga

**Affiliations:** 1 Department of Oral Medicine and Radiology MNR Dental College and Hospital, Narsapur road, Sangareddy (502294), Telangana, India

**Keywords:** CBCT artifacts, amalgam filling, foreign body

## Image in medicine

A 38 year old male patient presented to a radiology center for cone beam computed tomography (CBCT) and the working maxillofacial radiologist spotted a very peculiar finding - There was a large piece of metal in the mandible (size 0.5 cm X 0.5 cm). Here in we have provided a commentary on CBCT artifacts seen in this case along with a short explanation on the source of metal. Based on our observation the object in the mandible is certainly a metallic filling (See figure). This finding is based on the artifacts (acquisition artifacts) that were produced around the suspected material. Two types of artifacts were seen: 1. Scatter and 2. Beam hardening artifacts. Scatter occurs due to the diffraction of original beam after interaction with material. Scatter causes streak artifacts in the reconstruction (Blue arrows in panel A and C). Beam hardening artifacts were also found; they are more common. The lower energy rays suffer significant absorption when passing through the object. Higher the density and atomic number (metals) greater is the absorption. In the reconstruction image they are seen as darks streaks (Between blue arrows in panel B). Materials that often cause beam hardening include metallic restorations and titanium implants. Yes, even light metals such as titanium cause massive beam hardening and amalgam which is the material suspected in this case causes even greater hardening of beam. We strongly think that the metal must be amalgam. Some metal must have got displaced during the stage of condensation of filling in the tooth (most likely 36) just above the large radiolucent lesion. This is the most probable explanation. Unfortunately, this patient couldn't be followed up and the source of metal is still unknown leading to many speculations.

**Figure 1:**
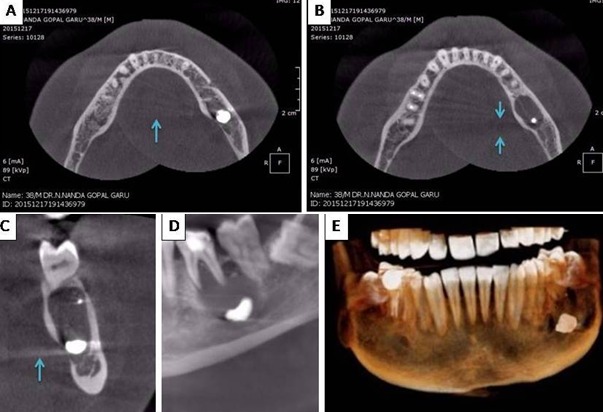
Blue arrows pointing scatter and beam hardening artifacts casted by a large metallic object in mandible (0.5x 0.5 cm)

